# The heme A synthase Cox15, as a target of redox-active 3-benzylmenadiones with antiparasitic activity

**DOI:** 10.1128/aac.01161-25

**Published:** 2025-12-10

**Authors:** Marcelo L. Merli, Claudia Serot, Cindy Vallières, Julia A. Cricco, Bogdan I. Iorga, Elisabeth Davioud-Charvet, Brigitte Meunier

**Affiliations:** 1 Instituto de Biología Molecular y Celular de Rosario (IBR), Consejo Nacional de Investigaciones Científicas y Técnicas (CONICET) - Universidad Nacional de Rosario (UNR) 28237, Rosario, Santa Fe, Argentina; 2 Université Paris-Saclay, CEA, CNRS, Institute for Integrative Biology of the Cell (I2BC) 27048, Gif-sur-Yvette, France; 3 Université Paris-Saclay, CNRS UPR 2301, Institut de Chimie des Substances Naturelles (ICSN) 27048, Gif-sur-Yvette, France; 4 UMR7042 CNRS-Unistra-UHA, Laboratoire d’Innovation Moléculaire et Applications (LIMA), European School of Chemistry, Polymers and Materials (ECPM) 105347, Strasbourg, France; The Children's Hospital of Philadelphia, Philadelphia, Pennsylvania, USA

**Keywords:** mitochondrial respiratory chain, drug mode of action, yeast model, antiparasitic drug

## Abstract

Chagas disease, caused by *Trypanosoma cruzi*, is a neglected parasitic infection. The very limited arsenal of anti-*T*. *cruzi* treatments calls for the development of new drugs. Recently, a library of 3-benzylmenadione derivatives was synthesized, with cruzidione being the most efficient and specific compound against the parasite. To decipher its mode of action, we used the yeast *Saccharomyces cerevisiae* as a model. Evidence pinpointed at the heme A synthase Cox15 as a primary target of cruzidione: (i) a mutation in Cox15 (i.e., S429F) renders the yeast cells highly sensitive to the drug, (ii) treatment with cruzidione led to the loss of cytochrome *c* oxidase, an enzyme that relies on heme A as an essential cofactor, and (iii) replacement of the yeast Cox15 by *T. cruzi* enzyme resulted in a high sensitivity to cruzidione. We then investigated the effect of cruzidione in *T. cruzi* and observed a significant reduction in the heme contents, most likely involving the inhibition of the heme A synthase. This, in turn, led to a decrease in O_2_ consumption by the parasite. Finally, using the yeast model, we showed that, similar to what we previously found for the antimalarial benzylmenadione plasmodione, NADH-dehydrogenase plays a key role in cruzidione bioactivation. We proposed that the reduced benzoylmenadione metabolites, produced by the reaction with NADH-dehydrogenase, act as Cox15 inhibitors. This study, through the identification of the mode of action of cruzidione, highlighted Cox15 as a novel target for antiparasitic drugs.

## INTRODUCTION

Parasitic diseases, such as Malaria and Chagas disease, caused by *Plasmodium sp*. and *Trypanosoma cruzi,* respectively, remain a major public health issue in many countries in the southern hemisphere. The limited arsenal of effective and nontoxic drugs to cure Chagas disease or the inefficacy of the antimalarial treatments to kill resistant parasites calls for the development of new drugs and the discovery of novel drug targets.

To that end, a chemical library of 3-benzylmenadiones (bMDs) was synthesized to introduce structural diversity and tested against both *P. falciparum* and *T. cruzi*. Promising compounds were discovered, such as plasmodione, which is active against *P. falciparum* (1–3), and cruzidione, which is active against intracellular *T. cruzi* amastigotes (4).

**Fig 1:**
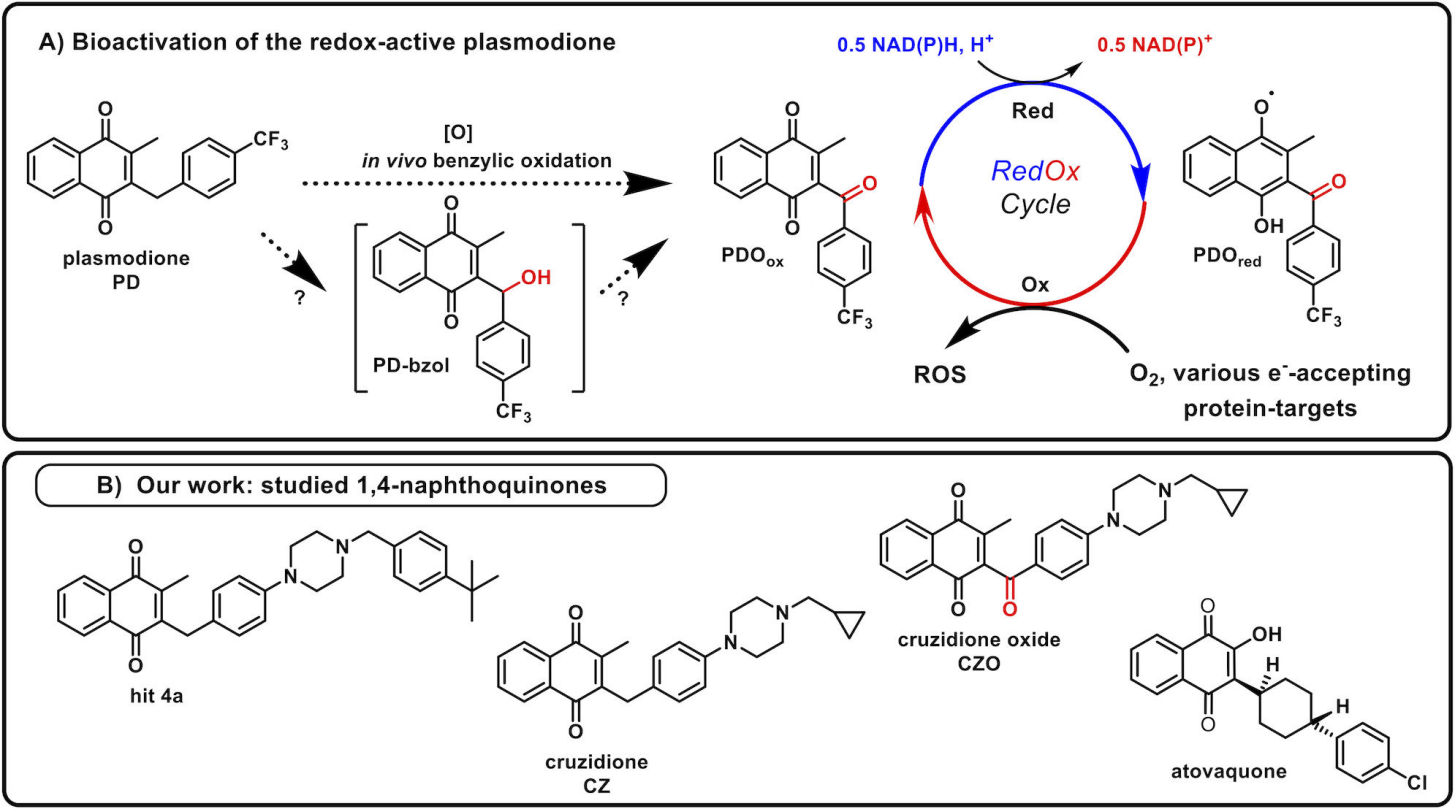
(**A**) Mechanism of action of the antimalarial plasmodione, simplified scheme (from [8]). Step 1: The benzylic oxidation of plasmodione (PD) generates the 3-benzoylmenadione metabolite (PDOox), possibly through a benzhydrol (PD-bzol) intermediate. Step 2: PDOox acts as a substrate of flavoenzymes (mainly NADH-dehydrogenase in yeast [9]) and is reduced, generating PDOred. Depending on the flavoenzyme target involved, the first one-electron transfer leading to the semi-quinone is proposed to be involved in the drug bioactivation, whereas the second one-electron transfer leads to the dihydronaphthoquinone PDOred. For the sake of clarity, only the one-electron-reduced naphthoquinone species are drawn. Step 3: PDOred transfers electrons to oxygen, leading to ROS production and possibly to other electron-accepting targets. Re-oxidized PDO binds again to and is reduced by flavoenzymes in a redox-cycling process. (**B**) Chemical structures of the anti-T. cruzi bMD derivatives: the hit **4a** and the lead cruzidione (CZ) (4), the benzoyl metabolite of CZ, named cruzidione oxide (CZO), and atovaquone (ATV).

Plasmodione (PD) (Fig. 1A) displays a fast-acting antimalarial activity against both early asexual and sexual stages of the parasite. Its mechanism involves the disruption of the redox balance within infected red blood cells (2). A proposed mode of action (MoA), based on *in vitro* assays (1, 3), suggests that PD mechanism begins with its benzylic oxidation, leading to the generation of 3-benzoylmenadione (PDO for plasmodione oxide), possibly through a benzhydrol (PD-bzol) intermediate (5, 6) (Fig. 1A). In *P. falciparum*, as in the yeast *S. cerevisiae* model, there is no cytochrome P450 that could perform the reaction. However, this step may be catalyzed by free or protein-bound hemes *via* ferryl species. Numerous hemoproteins (including hemoglobin) can indeed oxidize drugs (7).

Once formed, the highly oxidant PDO_ox_ acts as a subversive substrate of flavoproteins, initiating a redox cycling process that produces reactive oxygen species (ROS) (6). We showed that in yeast, the respiratory chain NADH-dehydrogenases (NDH) were the main enzymes for PDO redox-cycling (9).

We have proposed that PDO binds to NDH, maybe in a manner similar to menadione (10) and accepts electrons from NADH via the FAD co-factor (9). Once reduced, PDO_red_ transfers electrons to O_2_ to produce reactive oxygen species (ROS). The re-oxidized compound, PDO_ox_, binds again to and is reduced by NDH, creating a redox cycle. The continuous ROS production damages sensitive enzymes. In addition, the constant oxidation of NADH results in NADH/NAD imbalance, which could affect the NADPH/NADP balance, leading to increased oxidative stress and eventually growth arrest.

Supporting this, we found, in our yeast model, that PD treatment decreased the activity of aconitase, an enzyme sensitive to ROS. Also, when the superoxide dismutases Sod1 and Sod2 were absent, yeast cells became dramatically more sensitive to PD (9). This strongly indicated that the drug indeed caused ROS overproduction in the cells.

Cruzidione (CZ), which derives from the early hit **4a** (Fig. 1B), displays potent and specific anti-*T*. *cruzi* activity. Although the precise MoA of the anti-*T*. *cruzi* bMD remains largely unexplored, and initial yeast model studies suggest that CZ shares some mechanistic similarities with PD (4). Our *in vitro* assay showed that CZO, the benzoylmenadione derivative of CZ (Fig. 1B), reacts with yeast NDH and generates ROS, much like PDO. Furthermore, the absence of Sod1 and Sod2 severely increased the sensitivity of the yeast cells to CZ, indicating that CZ induces an intracellular oxidative stress.

However, this oxidative stress alone might not fully account for *T. cruzi* sensitivity to CZ. *T. cruzi* possesses robust antioxidant defenses, specifically the trypanothione reductase-trypanothione system, which can detoxify most of the ROS, including superoxide radicals and hydrogen peroxide, and nitrogen species. However, the parasite remains sensitive to menadione derivatives, suggesting that naphthoquinone radicals might interfere with essential processes in *T. cruzi* (11).

In order to uncover enzymes involved in the activity of the antiparasitic bMDs, we used the yeast model, *S. cerevisiae*. This organism is invaluable for studying the MoA of a drug as it offers powerful genetic and biochemical tools. In fact, *S. cerevisiae* has consistently proven to be instrumental in both drug discovery and the identification of drug targets (12).

We previously studied mutants with either deleted or overexpressed chosen genes (9) and selected and analyzed PD-resistant mutants (8). In this study, we shifted our focus to drug hypersensitive mutants to gain further insights. We specifically analyzed a mutant exhibiting increased sensitivity to PD and, with an even more marked effect, to the anti-*T*. *cruzi* bMDs.

The phenotype was caused by an amino acid substitution in the mitochondrial heme A synthase Cox15. Furthermore, we found that replacing yeast Cox15 with its *T. cruzi* homolog results in a severely increased sensitivity to the anti-*T*. *cruzi* bMDs **4a** and CZ.

We then monitored the effect of CZ on the growth, oxygen consumption, and heme level in *T. cruzi* epimastigotes and found that these parameters were strongly reduced upon drug treatment. These findings across both yeast and *T. cruzi* models strongly suggest that Cox15 is a key target for anti-*T*. *cruzi* bMDs like CZ that hold promises for future drug development against Chagas disease.

## MATERIALS AND METHODS

### Yeast methods

#### Growth media, yeast *S. cerevisiae* strains, and plasmids

The following growth media were used: YPD (1% yeast extract, 2% peptone, and 3% glucose), YPEth (1% yeast extract, 2% peptone, and 2% ethanol), YPG (1% yeast extract, 2% peptone, and 3% glycerol), YPGal (1% yeast extract, 2% peptone, 0.2% glucose, and 2% galactose), and CSM-uracil medium (0.7% yeast nitrogen base, 2% glucose, 2% agar, and 0.8 g/L of a complete supplement mixture minus uracil supplied by Bio 101 [San Diego, CA, USA]).

The parental strain AD1-9, kindly provided by M. Ghislain, UCL, Belgium, lacks several membrane transporters (α, *ura3, his1, Δyor1, Δsnq2, Δpdr5, Δpdr10, Δpdr11, Δycf1, Δpdr3, Δpdr15,* and *Δpdr1*), rendering the cells more sensitive to compounds added in the growth medium. The different mutants derived from AD1-9 and the plasmids used in this study are listed in Table 1. They were constructed in this study, with the exception of pNDE2, which was already available (9). Gene deletions and replacements resulted from homologous recombination of PCR products. For gene deletions, the *kanMX4* cassette was inserted in place of *COX15* or *NDE1*; for gene replacement, modified versions of *COX15* were inserted in the *COX15* genomic locus of the Δcox15 mutant, which was unable to grow on respiratory medium due to the absence of Cox15. The recombinant cells that harbored variant yeast *COX15* or *T. cruzi COX15* were selected for their respiratory growth competence.

**TABLE 1:** Yeast strains and plasmids

Strains	*COX15* version / mutation
AD1-9 (parental strain)	*COX15* WT
PDHS1	*cox15-S429F* (mutation found in the mutant issued from the selection of mutants hypersensitive to PD)
Δcox15	deletion of *COX15* (*cox15::kanMX4*)
cox15-S429F	S429F mutation in yeast *COX15*
cox15-S429A	S429A mutation in yeast *COX15*
cox15-Tc	*T. cruzi COX15* ORF replacing yeast *COX15* ORF
cox15-Tc-W125M	W125M mutation in *T. cruzi COX15* ORF replacing yeast *COX15* ORF
cox15-Tc Δnde1	*T. cruzi COX15* combined with the deletion of *NDE1* (*nde1:: kanMX4*)
Plasmids	
pCOX15	Yeast WT *COX15* cloned on YEp352 vector under the control of its promoter and terminator
pCOX15-S429F	derived from pCOX15
pCOX15-S429A	derived from pCOX15
pCOX15-E166L	derived from pCOX15
pCOX15-M165W	derived from pCOX15
pCOX15-Tc	*T. cruzi COX15* ORF cloned on YEp352 vector under the control of yeast *COX15* promoter and terminator
pCOX15-Tc-W125M	derived from pCOX15-Tc
pCOX15-Pf	*P. falciparum COX15* ORF cloned on YEp352 vector under the control of yeast *COX15* promoter and terminator
pCOX15-Hs	*Homo sapiens COX15* ORF cloned on YEp352 vector under the control of yeast *COX15* promoter and terminator
pNDE2	*NDE2* cloned on YEp352 vector under the control of its promoter and terminator

#### Growth assays-drug sensitivity tests

Drug sensitivity was assessed by monitoring the inhibition of yeast cell proliferation. Yeast cells were grown in 1 mL YPEth with increasing concentrations of drugs. When using ethanol as a carbon source, yeast cells solely rely on respiration to grow. Cultures were inoculated at an OD_600nm_ of 0.2 and incubated at 28°C with vigorous shaking for 3–4 days. OD_600nm_ was then measured. Each growth experiment was repeated at least twice. The data were averaged and plotted, with error bars representing standard deviation.

#### Cytochrome spectra of whole cells

The protocol was adapted from Laleve et al. (13). The cells were grown in 50 mL YPGal without or with drugs, with vigorous agitation. Galactose was used in these assays since this carbon source allows the cells to use both respiration and fermentation, permitting an estimation of cytochrome levels, including in respiratory-deficient mutants. The OD_600nm_ of the cultures was recorded after 28 h for normalization. The cells were harvested, dried on blot paper to remove excess liquid and increase the cell concentration, placed in a homemade cuvette, reduced with dithionite, and immediately frozen in liquid nitrogen. Cytochrome spectra were recorded with a Cary 400 (Varian, San Fernando, CA) spectrophotometer at liquid nitrogen temperature.

The magnitude of the cytochrome signals was measured at the wavelengths shown in Table 2.

**TABLE 2:** Wavelengths for cytochrome measurements

Cytochrome	Wavelength	Baseline
*b*	559 − 572 nm	540–572 nm
*c*	548 − 540 nm	540–572 nm
*aa_3_*	603 nm	594–612 nm

From these measurements, the ratios of the signals of cytochrome *c*/cytochrome *b* (c/b) and cytochrome *aa3*/cytochrome *b* (aa3/b) were estimated.

#### Isolation of hypersensitive mutants

AD1-9 cells, plated on YPD, were exposed for a short period of time (5–20 s) to UV to increase the mutation rate. After 2–3 days, the cells were replicated onto YPG plates and grown for an extra 2–3 days. The cells were then inoculated at an OD_600nm_ of 2 in YPEth and grown overnight. The cultures were then diluted 2-fold in fresh YPEth. Plasmodione was added at sub-inhibitory concentrations (2.5, 5, and 10 µM), and the cultures were incubated for 5.5 h with vigorous agitation. After the addition of nystatin at 50 µg/mL, the cultures were incubated for another hour. The cells were then washed, diluted, and plated on YPD medium. The survival rate was estimated to be 0.05%. The colonies were tested on YPG plates supplemented or not with 5 and 10 µM PD. Hypersensitive mutants were identified, subcloned, and re-tested. Whole genome sequencing and bioinformatics analysis were performed by the Next Generation Sequencing Core Facility of I_2_BC (www.i2bc.paris-saclay.fr/sequencing).

#### NADH-cytochrome *c* reduction measurements

Mitochondria were prepared as in the study of Lemaire et al. (14). Protein concentration was determined using the Bradford method. NADH-cytochrome *c* reductase activities were measured by monitoring the rate of reduction of cytochrome *c* spectrophotometrically at 550–540 nm over the 5-min time course. Measurements were performed at room temperature in 1 mL of 10 mM potassium phosphate pH 7, 2 mM KCN, and 20 µM cytochrome *c*. Mitochondria were added at 25 µg protein/mL. The reaction was initiated by the addition of 0.8 mM NADH.

### Structural analysis

A three-dimensional model of yeast Cox15 was predicted with the web interface of Protenix (15) starting from the protein sequence (UniProt code P40086) and the SMILES strings of heme O and heme B (without the Fe^2+^ ions), with the default parameters. The Fe^2+^ ions were then replaced in the predicted model from the structure of bacterial heme A synthase (PDB 6A2J). Structural analysis and image generation were performed with UCSF ChimeraX (16).

### *T. cruzi* methods

#### Growth media and strain

Tagged">*T*. cruzi epimastigotes (Dm28c strain) were routinely maintained at the mid-log growth phase by dilution every 3 days in fresh LIT medium (5 g/L liver infusion, 5 g/L bacto-tryptose, 68 mM NaCl, 5.3 mM KCl, 22 mM Na_2_HPO_4_, and 0.4% glucose, pH 7.4) supplemented with 10% heat-inactivated fetal bovine serum and 5 μM hemin (LIT-10% FBS-5 μM hemin) at 28°C. Fetal bovine serum was obtained from Internegocios S.A. (Buenos Aires, Argentina) and heat-inactivated at 56°C for 30 min. Hemin stock solution (1 mM) was prepared in 50% EtOH, 0.01 M NaOH.

#### Growth assays-drug sensitivity tests

The epimastigotes were diluted at a concentration of 5 × 10^6^ parasites/mL and challenged to grow in LIT-10% FBS-5 μM hemin supplemented with 0, 0.625, 1.25, 1.875, 2.5, 3.75, 5, 10, and 15 µM CZ (three biological replicas per condition). The final number of cells was determined after 3 days using a Neubauer counting chamber. Data analysis for IC_50_ was performed using the GraphPad Prism 5 software. A graph of the log of concentration vs. number of epimastigotes was performed and adjusted using “log(inhibitor) vs. response − Variable slope” analysis.

#### Oxygen uptake measurements

The cells were diluted at a concentration of 5 × 10^6^ parasites/mL and challenged to grow in LIT-10% FBS-5 μM hemin supplemented with 0, 1.25, and 2.5 µM CZ (three biological replicas per condition). After 3 days, the oxygen consumption rates of epimastigotes (O_2_ nmoles mL^−1^ min^−1^ 10^6^ cells) were quantified from the linear response, using a Clark electrode connected to a 5300 Biological Oxygen 528 Monitor (Yellow Springs Instrument Co.), as previously described (17).

#### Heme A and B quantification

The epimastigotes were diluted at a concentration of 5 × 10^6^ parasites/mL and challenged to grow in LIT-10% FBS-5 μM hemin or in LIT-10% FBS without hemin, supplemented with 0, 1.25, and 2.5 µM CZ (at least two replicas per condition). After 3 days, the cells were collected (2,000–4,000 × 10^6^ parasites/sample), hemes B and A were extracted by the acetone–HCl extraction, and their concentrations were quantified from the reduced-oxidized differential spectrum as described previously (17). The heme B and heme A molar extinction coefficients are 23.98 (at 557 nm) and 25.02 (at 588 nm) mM^−1^ cm^−1^, respectively (18).

## RESULTS

### A yeast Cox15 mutant with increased sensitivity toward antiparasitic bMDs

In order to identify the primary targets of bMDs, we generated mutants by UV exposure and selected the ones hypersensitive to the drugs. The strategy is based on the counter-selection of growing cells by exposure to nystatin in the presence of the drug of interest at concentrations that are sub-inhibitory for the WT population. At these concentrations, the growth of hypersensitive mutants is inhibited, protecting the cells against nystatin-induced cell death, whereas the growing cells are killed upon addition of nystatin. As a consequence, the cultures are enriched in hypersensitive mutants.

Here, we used PD to select hypersensitive mutants, and we identified eight mutants with increased PD sensitivity. Growth assays were then performed to confirm the sensitivity of the mutants to PD. Growth competence and sensitivity to the anti-*T*. *cruzi* bMDs CZ and **4a**, and to the hydroxy-1,4-naphthoquinone atovaquone, inhibitor of complex III, were also assessed. To do so, the mutants were cultivated in YPEth medium supplemented with the compounds, and the OD_600nm_ was measured after 3 days (data not shown). One mutant, named PDHS1, presented a particularly interesting phenotype as it showed a WT growth competence (i.e., reaching the same OD_600nm_ as its parental strain), a hypersensitivity to PD, CZ and **4a**, but a sensitivity to atovaquone comparable with the WT strain (data not shown), indicating that the hypersensitivity observed might be specific to bMDs and not caused by a more general mechanism, for example, a decreased activity of multidrug resistance system that would have conferred hypersensitivity to both bMDs and atovaquone (19).

**Fig 2:**
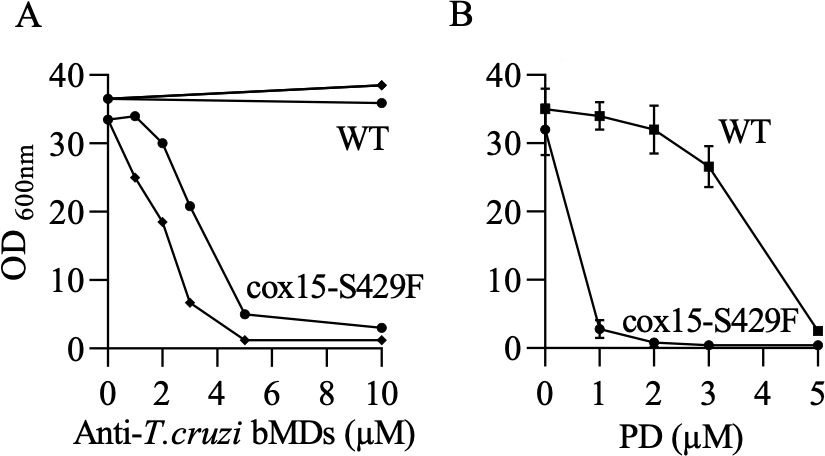
Growth sensitivity to bMDs of WT and cox15-S429F. Mutant cox15-S429F and its parental strain (WT) were grown in YPEth medium with various concentrations of drugs. OD600 nm was measured after 3 days. (**A**) The effect of anti-*T. cruzi* bMDs, 4a (◆), and CZ (●); (**B**) the effect of PD. The values in panel B are means ± SD from two measurements.

In order to identify the mutation causing the hypersensitivity, the whole genome of PDHS1 was sequenced. The sequence of the mutant was compared with the sequence of its parental strain, AD1-9. Approximately 80 possible changes compared with the parental strain were observed (40 in ORFs). The apparent large number of changes was previously seen in yeast-resistant mutants issued from the selection on PD-supplemented medium (8). Among the changes found in the PDH1 sequence, the most likely mutation causing the phenotype was identified on the basis of the function of the protein encoded by the mutated gene. The presence of the mutation was confirmed by PCR and subsequent sequencing of the gene of interest using independent DNA preparations. The mutation resulted in an amino acid substitution, namely S429F, in the heme A synthase Cox15. The mutation was then introduced in the parental AD1-9 strain by homologous recombination of a PCR product. The resulting mutant, named cox15-S429F, showed WT growth competence and displayed a strong sensitivity to anti-*T*. *cruzi* bMDs **4a** and CZ with an approx. IC_50_ (midpoint inhibitory concentration) of 3–4 µM (Fig. 2A), whereas the parental WT strain was unsensitive to those drugs (tested to up to 50 µM). Cox15-S429F also showed an increased sensitivity to PD (Fig. 2B).

### Loss of cytochrome *c* oxidase caused by bMDs in yeast

Cox15 catalyzes the synthesis of heme A, an essential cofactor of the cytochrome *c* oxidase, the terminal enzyme of the respiratory chain. Heme A is uniquely used by that enzyme. Therefore, the absence of Cox15 or the inhibition of its activity should result in the absence of cytochrome *c* oxidase.

**Fig 3:**
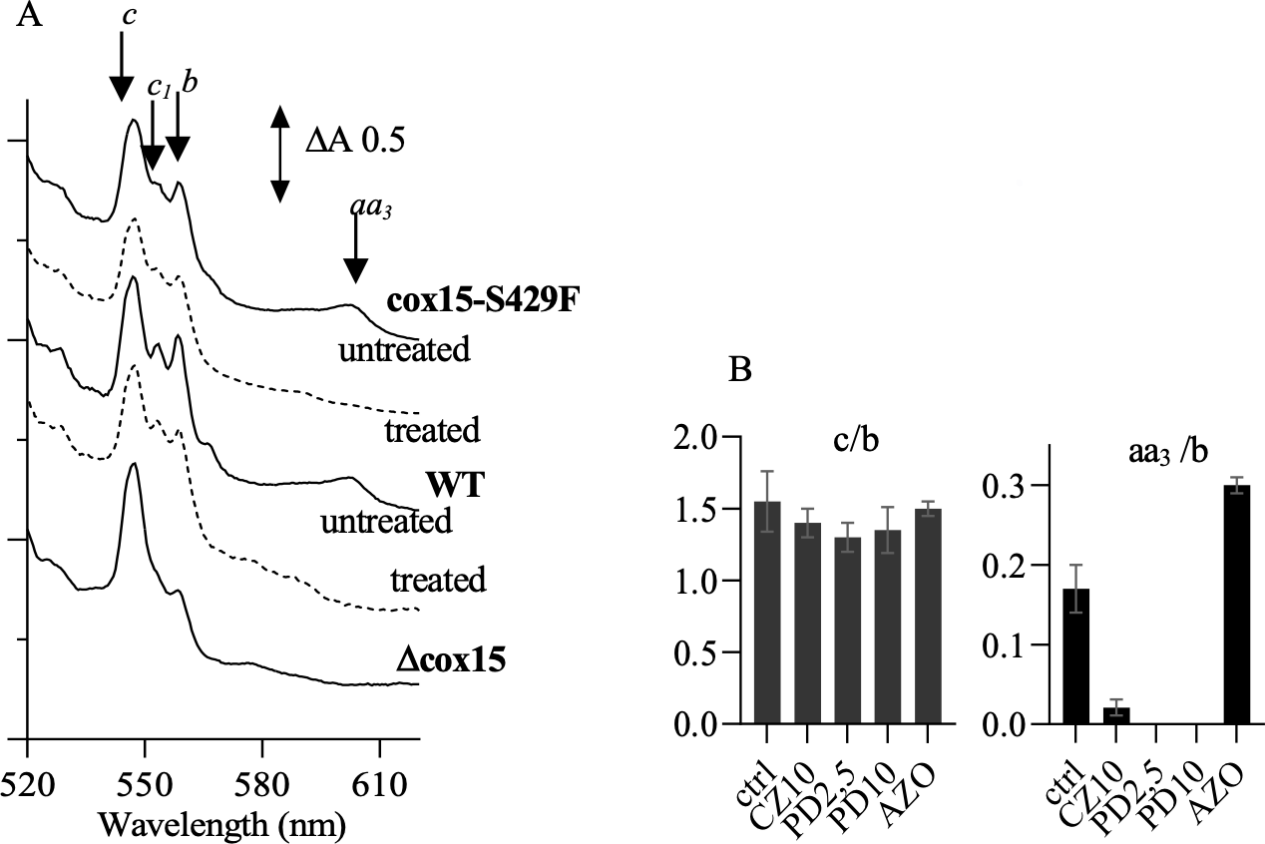
Effect of bMD treatment on cytochrome spectra in whole cells. (**A**) The cells were cultured for 28 h in YPGal medium with vigorous agitation without (solid lines) or with bMDs (dotted lines). Cox15-S429F was treated with 10 µM CZ, and WT with 10 µM PD. (**B**) Ratio cytochrome *c* /cytochrome *b* (c/b) and cytochrome *aa3*/cytochrome *b* (aa3/b) were measured in Cox15-S429F cells untreated (ctrl) and treated with 10 µM CZ (CZ10), 2.5, and 10 µM PD (PD2.5 and PD10), and 1 µM azoxystrobine (AZO). The values are means ± SD from two replicate measurements.

We monitored the cytochrome levels in mutant cox15-S429F, in a mutant lacking Cox15, Δcox15, and their parental strain (WT). The cells were grown in YPGal medium. With galactose as a carbon source, yeast cells can use both respiration and fermentation to grow. In Δcox15, the loss of cytochrome *c* oxidase results in the complete absence of respiratory growth (no alternative oxidase being present in *S.cerevisiae*) and the mutant relies on fermentation for growth. The absorption spectra of cox15-S429F and WT showed the four expected peaks corresponding to the cytochrome *c*, cytochromes *c*_1_ and *b* of the *bc*_1_ complex (or complex III), and cytochrome *aa*_3_ of cytochrome *c* oxidase (or complex IV) (Fig. 3A solid lines). By contrast, the cytochrome *aa*_3_ signal was absent in Δcox15, as expected. The increased cytochrome *c* level compared with cytochrome *b* in that mutant was not unusual, as we had previously observed that feature in mutants lacking cytochrome *c* oxidase.

We then assessed the effect of bMD treatment on cytochrome levels in cox15-S429F. When cultured in the presence of the anti-*T*. *cruzi* CZ, the level of cytochrome *c* oxidase dramatically decreased (Fig. 3A dotted line). The same experiment was performed with PD and with the complex III inhibitor azoxystrobin (AZO). Figure 3B shows the effect of the treatment with CZ, PD, and AZO on cytochrome *c* and cytochrome *aa_3_* (presented as ratio *c*/*b* and *aa3*/*b*, respectively). In the conditions of the assays, the final OD_600nm_ of the treated cultures was decreased by nearly half, compared with the untreated culture. Treatment with CZ and PD resulted in a severe loss of cytochrome *c* oxidase (cytochrome *aa*_3_), whereas exposure to AZO had the opposite effect, indicating that the loss of cytochrome *c* oxidase signal observed with both bMDs was due to the drugs and not a result of growth inhibition. The effect of PD treatment on the WT cells was also monitored. It resulted in a severe decrease in cytochrome *aa*_3_ (Fig. 3A, dotted line).

### High sensitivity to anti-*T*. *cruzi* bMDs of yeast cells expressing *T. cruzi* Cox15

As the amino acid substitution S429F in Cox15 resulted in a severe sensitivity to anti-*T*. *cruzi* bMDs, we tested the effect of the expression in yeast of *T. cruzi COX15* and for comparison of human and *P. falciparum COX15*.

To that end, *COX15* ORFs from *T. cruzi,* human, and *P. falciparum* were cloned into a multicopy vector and expressed under the control of the yeast *COX15* promoter and terminator. Mutant Δcox15 was transformed by the plasmids, and the resulting strains were first tested for their respiratory growth competence. *T. cruzi* and human Cox15 were functional in yeast as the culture of the cells expressing the exogenous Cox15 reached the same OD_600nm_ as the control, that is, mutant Δcox15 expressing yeast *COX15* cloned in the multicopy vector. This was expected from published reports (20, 21). By contrast, *P. falciparum* Cox15 seemed unable to replace the yeast enzyme as the cells remained respiratory growth-deficient, and therefore, their sensitivity to bMDs could not be assessed.

**Fig 4:**
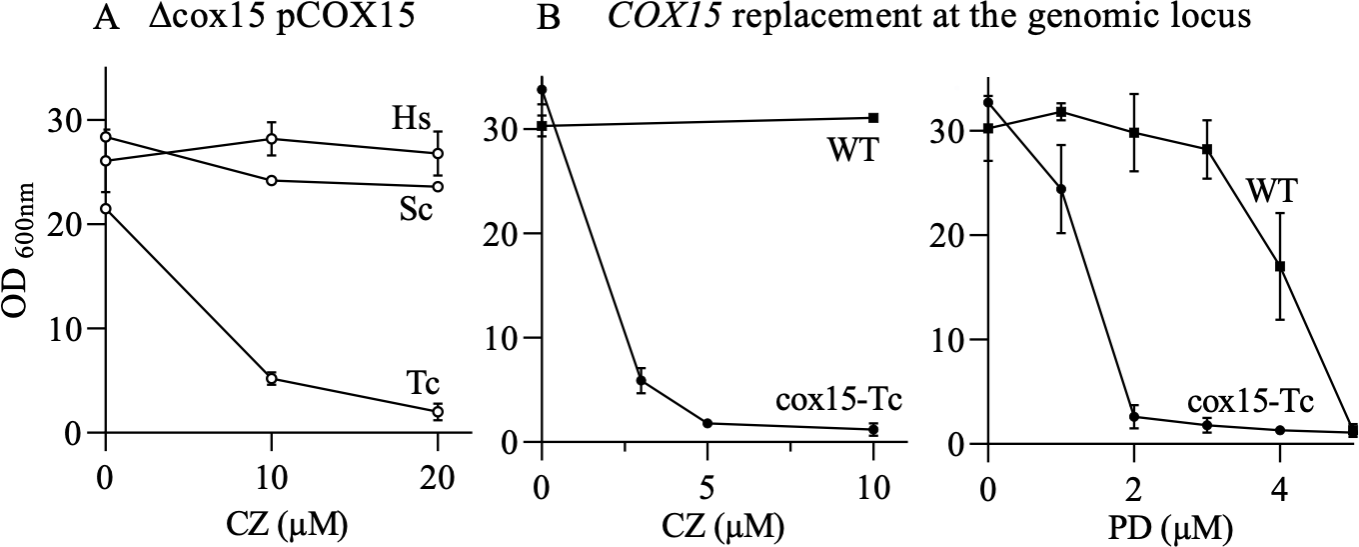
Growth sensitivity to bMDs of mutants expressing exogenous *COX15*. (**A**) Effect of the expression of *COX15* from *S. cerevisiae* (Sc), *T. cruzi* (Tc), and *H. sapiens* (Hs). Mutant Δcox15 was transformed with a plasmid harboring yeast *COX15* (control, Sc) or *COX15* from the two other species under the control of yeast *COX15* promoter and terminator, and (**B**) the effect of the expression of *T. cruzi COX15* ORF replacing yeast *COX15* ORF at the genomic locus (cox15-Tc). The cells were grown in YPEth medium with various concentrations of CZ and PD. OD_600nm_ was measured after 3 days. The values are means ± SD of two replicate measurements.

The anti-*T*. *cruzi* bMD sensitivity of the yeast cells expressing *T. cruzi* and human *COX15* was then tested. The presence of *T. cruzi* Cox15 (TcCox15) rendered the cells sensitive to CZ, whereas the presence of the human Cox15, as that of the yeast Cox15, resulted in cells unaffected by CZ (tested at up to 20 µM) (Fig. 4A). The same results were obtained with compound **4a** (Fig. S1A).

In order to confirm the finding, yeast *COX15* ORF was replaced by *T. cruzi COX15* ORF at the genomic locus. The resulting mutant named cox15-Tc was then tested for its sensitivity to bMDs (Fig. 4B). The mutant displayed a WT growth, a 2-fold increase in PD sensitivity (IC_50_ mutant/IC_50_ WT), and >20-fold increase in sensitivity to anti-*T*. *cruzi* compounds. Cox15-Tc showed >80% growth inhibition in the presence of 5 µM CZ. The same data were obtained with **4a** (Fig. S1B). By contrast, as mentioned above, the parental strain was unaffected at 50 µM anti-*T*. *cruzi* bMDs. For comparison, the effect of the anti-malaria compound artesunate was also tested. Cox15-Tc, as cox15-S429F, presented a WT sensitivity (Fig. S1C).

### Effect of CZ on heme levels in the parasite *T. cruzi*

Replacement of the native heme A synthase by *T. cruzi* Cox15 (TcCox15) resulted in yeast cells highly sensitive to CZ, inferring that the drug interferes with the parasite enzyme (whereas CZ would be poorly effective on the yeast and the human enzymes). Therefore, we tested whether CZ might inhibit TcCox15 in *T. cruzi* and decrease heme A level in the parasite.

**Fig 5:**
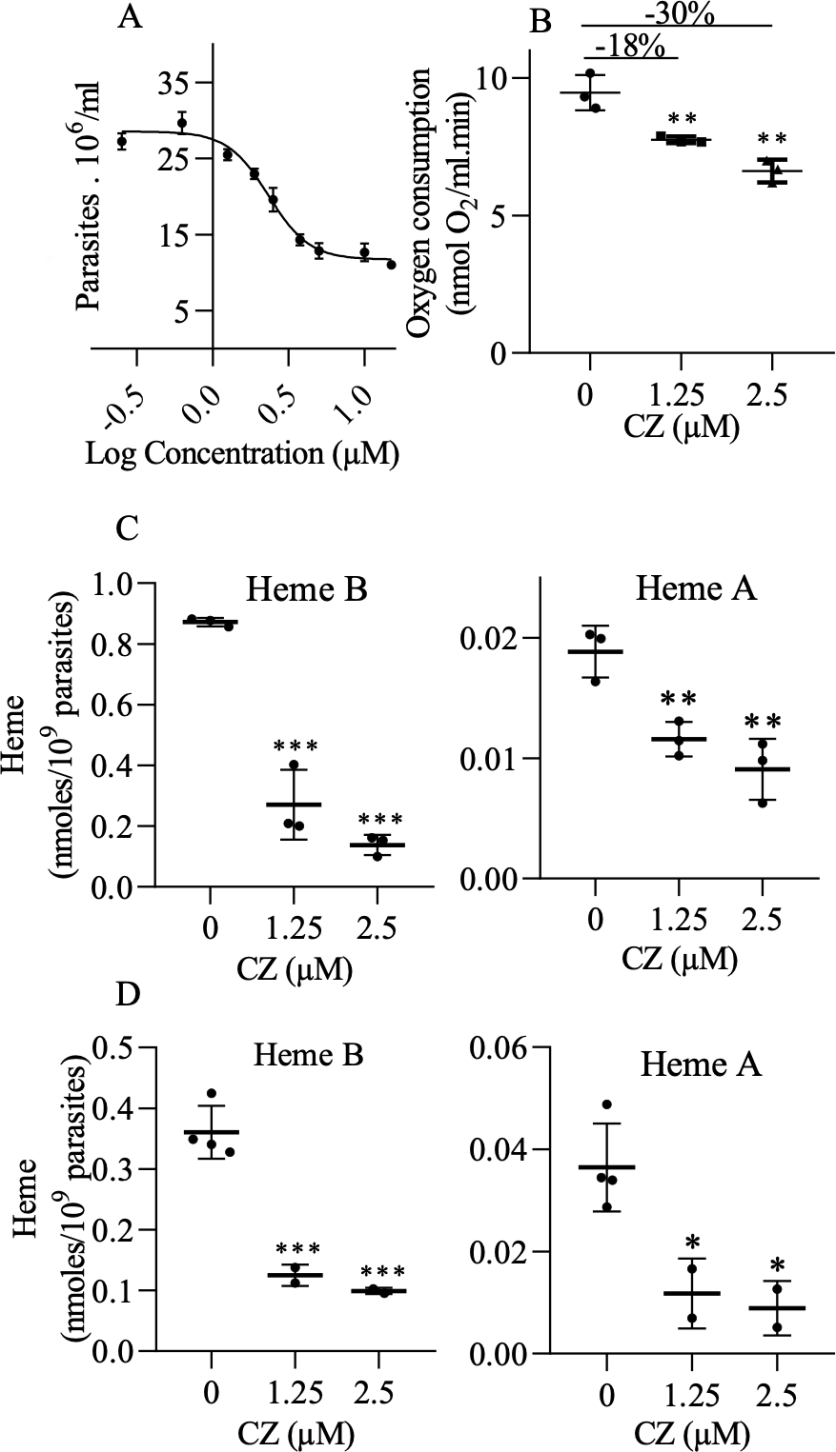
Effect of CZ treatment in *T. cruzi*. (**A**) Effect of CZ on *T. cruzi* growth. Epimastigotes were diluted at a concentration of 5 × 10^6^ parasites/mL and cultured with 0, 0.625, 1.25, 1.875, 2.5, 3.75, 5, 10, and 15 µM CZ. The cell numbers were determined after three days and graphed vs. Log of concentration. The measurements were repeated at least twice and the data averaged (●). The error bars represent standard deviations. The line represents the adjustment using “log(inhibitor) vs. response − Variable slope” analysis. (**B**) Effect of CZ on oxygen consumption. The cells were diluted at a concentration of 5 × 10^6^ parasites/mL and cultured with 0, 1.25, and 2.5 µM CZ. After 3 days, the oxygen consumption rates of epimastigotes (O_2_ nmoles mL^−1^ min^−1^ 10^6^ cells) were quantified. (**C and D**) Effect of CZ on hemes B and A levels. Epimastigotes were routinely maintained in fresh LIT medium (LIT-10% FBS-5 μM hemin). For heme determination, the cells were diluted at a concentration of 5 × 10^6^ parasites/mL and challenged with 0, 1.25, and 2.5 µM CZ in the same medium (**C**) or in LIT medium without hemin (**D**). After 3 days, heme B and A were extracted and quantified. The points represent independent measures and are the average of two technical replicates. The measurements were repeated at least twice and the data averaged (line). The error bars represent standard deviations. The differences compared with the control were determined using one-way ANOVA and Dunnett post-hoc test. Significance is shown as asterisks: **P < 0.01 and ***P < 0.001.

First, we tested the effect of CZ on epimastigote growth and estimated an IC_50_ of 2.3 µM (Fig. 5A), which was close to the IC_50_ value of 3.6 µM previously reported for the intracellular amastigotes (4). To test a potential effect of CZ on TcCox15, we first monitored the effect of the drug on oxygen consumption at both growth inhibitory concentration (i.e., close to IC_50_ value) and growth sub-inhibitory concentration; higher concentrations of CZ might affect non-specifically diverse metabolic pathways including the respiration. As shown in Fig. 5B, CZ treatment decreased oxygen consumption in a dose-dependent manner, with 18% and 30% reduction at 1.25 and 2.5 µM, respectively.

We then monitored the effect of CZ on heme A level in epimastigotes grown in standard medium (with the addition of 5 µM hemin) or without the addition of hemin for 3 days. We included the second condition to obtain a better resolution of heme A signal, as higher heme B signal distorts heme A band affecting the quality of the quantification. CZ treatment significantly reduced the levels of heme A in the parasites but also heme B (Fig. 5C and D). For instance, at sub-inhibitory concentration (i.e., 1.25 µM), heme B and heme A levels were reduced by ~70% and ~50%, respectively, demonstrating a strong effect of CZ on heme metabolism.

In this study, we used epimastigotes to perform the analysis. It is most likely that CZ could act in the same manner in amastigote, which would be the most relevant stage to validate compounds against *T. cruzi*. As mentioned above, our results have shown that the IC_50_ of CZ was similar in both stages (epimastigotes and amastigotes, 3.6 µM (4) and 2.3 µM, respectively). Also, we have previously demonstrated that TcCox15 activity is important for the parasite replication in amastigotes and epimastigotes stages (17).

### Investigating the interaction between CZ and Cox15 in the yeast model

We observed that yeast cells harboring the mutated Cox15 Cox15-S429F or TcCox15 were highly sensitive to CZ (and **4a**). Using the yeast model, we then attempted to explore the interaction between CZ and Cox15. First, we explored the structure of Cox15 and the location of the mutation causing CZ hypersensitivity to identify the possible binding region of bMDs.

#### Possible binding site of bMDs on Cox15

Heme A derives from heme B by the sequential actions of the heme O synthase (Cox10), which mediates the farnesylation of a vinyl group to yield the heme O, and heme A synthase (Cox15), which converts heme O to heme A through oxidation of its C8-methyl group (see (22–24) and refs within). The catalytic mechanism of the heme A synthase is not fully understood. It is likely to involve radical reactions. In the first step of a proposed mechanism (23), a single-electron mediated oxidation of heme O generates a heme radical, which produces an ester-crosslink of the heme C8-methyl group with the carboxylate group of a glutamate (E166 in yeast Cox15). A heme I intermediate is generated following hydrolysis. In the second step, another single-electron-mediated oxidation takes place. The reaction is then pursued to yield heme A. An alternative mechanism (24) proposed four one-electron transfer steps that are catalyzed by high-valent iron-oxo species and also invoke the formation of a carbon-centered radical at heme C8. In both proposed mechanisms, the highly conserved glutamate (E166 in yeast) participates in the reaction either by stabilizing the carbon-centered radical or by transiently forming an ester crosslink to this carbon.

**Fig 6:**
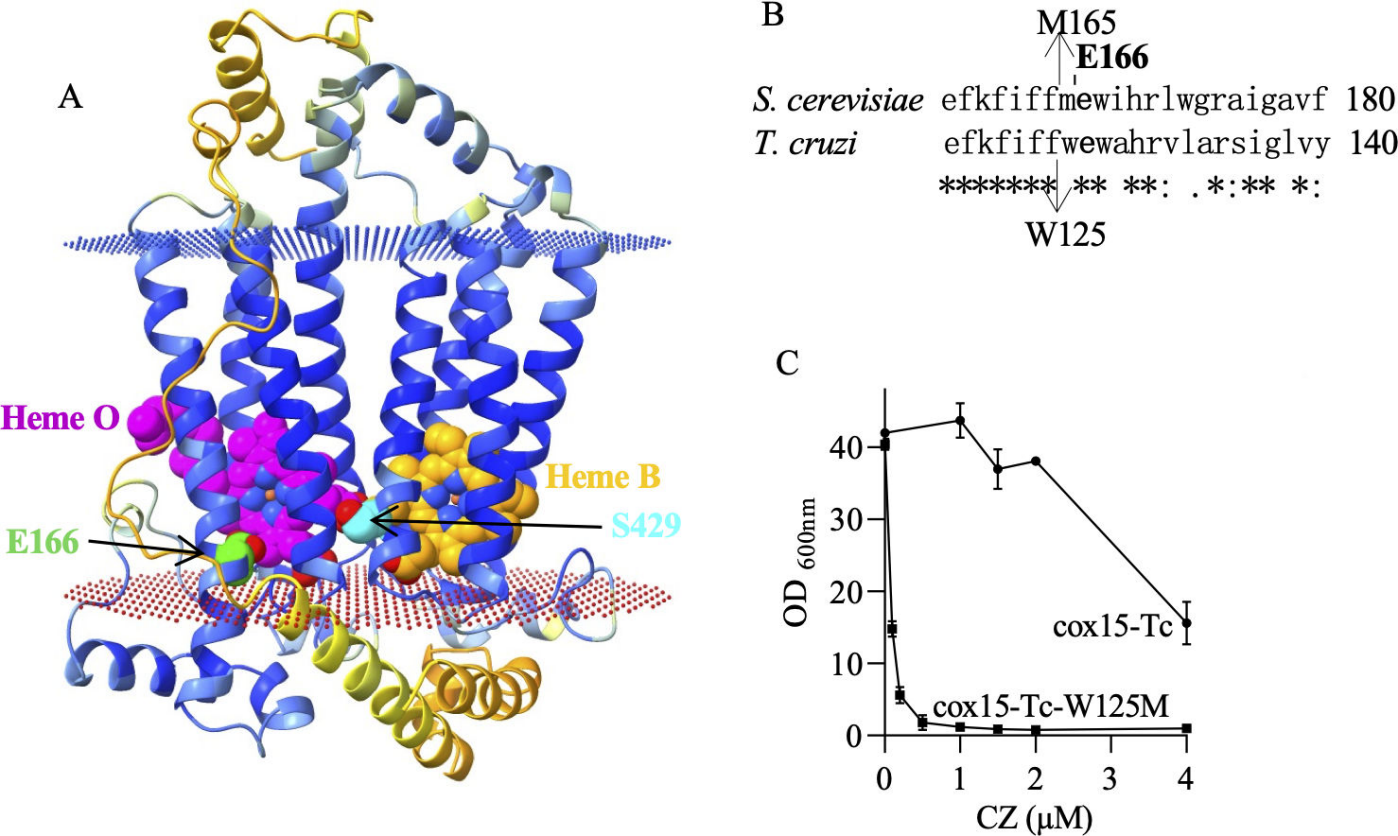
(**A**) Complete three-dimensional model of yeast Cox15. The secondary structure is colored by the predicted local distance difference test (pLDDT) value. The color scale represents confidence level: orange, very low; yellow, low; light blue, high; and blue, very high. The substrate heme O, the co-factor heme B, and residues E166 and S429 are shown in CPK representation and colored in magenta, orange, green, and cyan, respectively. The dots represent the membrane extremities as predicted by the PPM server (https://opm.phar.umich.edu/ppm_server). (**B**) Comparison of yeast and T. cruzi Cox15 sequence adjacent to residue E166; (**C**) Sensitivity to CZ of yeast mutant cox15-Tc-W125M compared to cox15-Tc. The cells were grown in YPEth medium with increasing concentrations of CZ. OD_600nm_ were measured after 3 days. The values are means ± SD of two replicate measurements.

The structure of the bacterial enzyme has been solved (23). However, the structure of the mitochondrial Cox15 is not available. We thus generated a complete model of yeast Cox15 using Protenix and the available bacterial heme A synthase structure (PDB 6A2J) (Fig. 6A).

In this model, the heme C8-methyl that is converted to formyl during the reaction is facing E166, the conserved glutamate proposed to be a key residue for heme A synthase activity. It was previously reported that the mutations E166A and E166D in yeast resulted in the absence of respiratory growth and of heme A (24). E166L, generated in this study, also abolished the respiratory growth (Fig. S2A).

Residue S429 (mutated to a phenylalanine in the CZ sensitive mutant cox15-S429F) is situated in close proximity to the two hemes (the substrate heme O and the cofactor heme B). The replacement of serine by phenylalanine could stabilize the binding or facilitate the access of the bMDs into Cox15 either directly or by inducing a local conformational change. We found that the replacement of S429 by an alanine had no effect on growth competence or sensitivity to bMDs (Fig. 2B and C), suggesting that the sensitivity observed in S429F was caused by a steric change induced by the bulky phenylalanine or by a stabilization of bMD binding *via* aromatic interactions or cation-π interactions (with the protonable piperazine nitrogen atom) and not by the loss of the polar residue serine. In the *T. cruzi* enzyme, S429 is replaced by a similar residue, that is, a threonine.

We then explored sequence differences that might contribute to TcCox15 sensitivity to CZ, focusing on aromatic residues. Interestingly, the residue adjacent to the conserved glutamate E166, M165 in yeast Cox15 is a tryptophan in TcCox15 (W125 in *T. cruzi* sequence) (Fig. 6B). We introduced the amino acid substitution W125M in TcCox15 and M165W in yeast Cox15 and monitored the sensitivity to CZ. M165W had little effect (Fig. S2D). By contrast, W125M had a significant impact. Unexpectedly, the mutation markedly increased, and not decreased, the sensitivity to CZ (Fig. 6C). Thus, the presence of a tryptophan adjacent to the conserved glutamate cannot explain the sensitivity to CZ of mutant cox15-Tc. Most likely, a combination of several changes compared with the yeast sequence renders *T. cruzi* enzyme sensitive to CZ. In TcCox15, the aromatic side chain of residue W125 might limit the access of the compound to its target site, whereas a methionine would facilitate its access.

Although more work is needed to identify the possible binding site of bMDs, our observations point toward bMDs interacting with Cox15 at the region of the substrate heme O and of the essential glutamate, thus interfering with the catalytic process.

#### Reduced 3-benzoylmenadione metabolites as possible inhibitors of Cox15

We hypothesized that reduced 3-benzoylmenadione CZO_red_ (and PDO_red_) reacts with heme radicals during Cox15 catalytic activity and inhibits the synthesis of heme A. We argue that if CZO_red_ acts as an inhibitor of TcCox15, the sensitivity of the yeast cells would depend on the intracellular level of that compound. CZO_red_, following the mechanism for PD bioactivation (Fig. 1), would be produced by the NADH-dehydrogenases (NDH).

**Fig 7:**
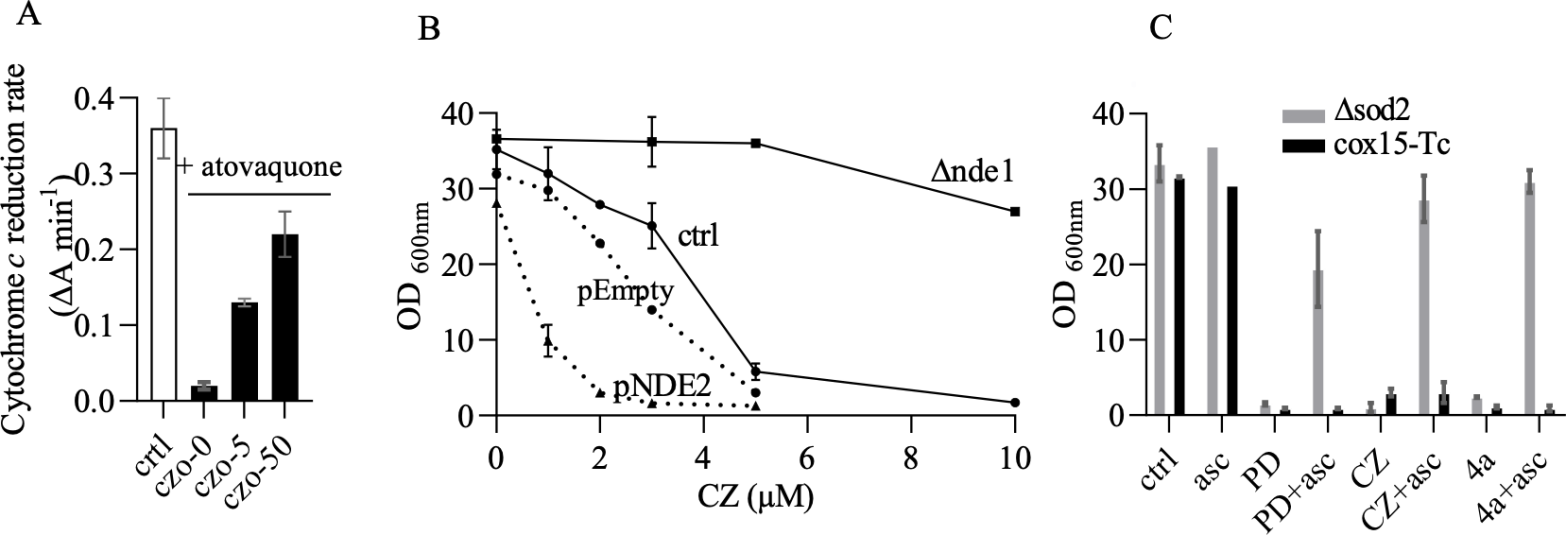
(**A**) NADH-cytochrome *c* reductase activity induced by CZO. The rates of cytochrome *c* reduction were measured spectrophotometrically at 550–540 nm. Mitochondria were added at around 25 µg protein mL^−1^. The reactions were initiated by the addition of 0.8 mM NADH. The rates are shown in ΔA_550-540_ min^−1^. White, control rate without the addition of atovaquone; black, rates in the presence of atovaquone; and CZO-5 and CZO-50, rates after the addition of 5 and 50 µM CZO. (**B**) Effect of NADH-dehydrogenase level on cox15-Tc growth sensitivity to CZ. Cox15-Tc (ctrl), cox15-Tc with the deletion of *NDE1* (Δnde1), cox15-Tc transformed with an empty vector (pEmpty), and cox15-Tc transformed with the multicopy vector bearing *NDE2* (pNDE2) were grown in YPEth with increasing concentration of CZ. The OD_600nm_ was measured after 3 days. (**C**) Effect of ascorbate to cultures treated with bMDs: comparison between Δsod2 (gray) and cox15-Tc (black). Cells were grown in YPEth medium with 2 µM PD, 10 µM CZ, or 10 µM **4a**, and with or without 8 mM ascorbate (asc). Control (ctrl), cultures without asc or bMDs. OD_600nm_ was measured after 3 days. The values are means ± SD of two replicate measurements.

First, we confirmed that CZO_ox_ can act as a substrate of NDH initiating redox-cycling reactions that produce superoxide anion radicals as already reported (4). To do so, we monitored the rate of NADH-driven cytochrome *c* reduction using purified mitochondria (Fig. 7A). In the control measurement (white column), cytochrome *c* was reduced by the combined activity of NDH and complex III. Addition of atovaquone (black) inhibited complex III, and the rate of cytochrome *c* reduction dropped to approximately 5% of the control rate. Addition of CZO at 5 and 50 µM restored the reduction rate to approximately 40% and 70% of the control rate, which could be explained by cytochrome *c* being reduced in a complex III-independent manner by the superoxide anions generated by the reaction of CZO with NDH, as shown for PDO (8). It is indeed known that superoxide anions can directly reduce cytochrome *c* (25).

Second, we tested the effect of increasing or decreasing NDH level on the sensitivity of the yeast cells harboring TcCox15 (i.e., mutant cox15-Tc) to the anti-*T*. *cruzi* bMDs. To that end, we deleted the *NDE1* gene encoding the main NDH or transformed the cells with a multicopy vector bearing *NDE2*. We previously showed that the deletion of *NDE1* in the parental AD1-9 strain resulted in a 4-fold to 5-fold decrease in NDH activity, whereas the overexpression on *NDE2* led to a 2-fold increase in NDH activity (see Fig. 4B in [9]). The new constructs, named cox15-Tc Δnde1 and cox15-Tc pNDE2, were then tested for their sensitivity to the anti-*T*. *cruzi* bMDs. As shown in Fig. 7B, the deletion of *NDE1* resulted in a decreased sensitivity to CZ, whereas the overexpression of *NDE2* resulted in an increased sensitivity. The same results were obtained with **4a**. The same tests were also performed with Cox15-S429F and similar results were obtained (not shown). Thus, the sensitivity of cox15-Tc (and of cox15-S429F) depends on the level of NDH and of the subsequent production of CZO_red_.

According to the MoA presented in Fig. 1, CZO_red_ reacts with oxygen to produce ROS, which would damage sensitive cell components and, eventually, could lead to growth inhibition. However, the increased ROS production might not be the sole, or even the primary cause of the growth defect observed upon CZ treatment.

We hypothesized that CZO_red_ interferes with Cox15 activity, leading to the inhibition of heme A synthesis. Heme A shortage, resulting from Cox15 dysfunction, would in turn result in cytochrome *c* oxidase deficiency and growth defect. CZ treatment would have (at least) two damaging outcomes (i) the generation of ROS (and its pleiotropic impacts) and (ii) the inhibition of Cox15 by CZO_red_. The same effects would be obtained with **4a** and also PD.

We thus attempted to deconvolute the effects. In order to get clues on the impact of ROS generation in bMD-induced growth inhibition, we tested the effect of ascorbate in cultures treated with CZ, **4a**, and PD. We compared the mutants Δsod2 and cox15-Tc, both highly sensitive to bMDs (Fig. 7C). In the conditions of our cultures, ascorbate was previously shown to act as an anti-oxidant and to rescue the growth of mutant Δsod2 treated with primaquine (26).

Addition of ascorbate rescued the growth of Δsod2 inhibited by bMDs, but not the growth of cox15-Tc. The antioxidant effect of ascorbate compensated for the absence of Sod2 by, presumably, decreasing the level of damaging ROS. It did not protect cox15-Tc against bMDs. Thus, the growth defect of cells with native yeast Cox15 and lacking Sod2 would be caused by bMD-induced ROS overproduction. The growth defect of cells with TcCox15 and a functional Sod2 would be mainly caused by the loss of cytochrome *c* oxidase, resulting from the inhibition of the heme A synthase by the bMDs that possibly act via their reduced 3-benzoylmenadione metabolites.

## DISCUSSION

Based on the data presented here using the yeast model, it is likely that the anti-*T*. *cruzi* bMDs share, at least partially, the same MoA with PD (Fig. 1). The pro-drugs would be metabolized producing 3-benzoylmenadione e.g., PDO from PD and CZO from CZ.

The 3-benzoylmenadione metabolites would act as subversive substrates of NDH. The reduced 3-benzoylmenadione could then react in the following two ways: (i) with oxygen to produce superoxide anions, which would damage ROS-sensitive cell components, and (ii) with Cox15 to inhibit heme A synthase, which by consequence would impact cytochrome *c* oxidase, respiratory function, and growth.

The mutational analysis pinpointed the heme A synthase Cox15 as bMDs target. Amino acid substitutions in yeast Cox15 and the replacement of the yeast by *T. cruzi* enzyme resulted in an increased sensitivity to bMDs in growth assays. In addition, bMD treatment caused a clear decrease in cytochrome *c* oxidase that requires heme A as an essential co-factor. We suggest that bMDs, more specifically, their reduced benzoylmenadione metabolites bind at the region of Cox15 substrate heme O and of the essential glutamate and inhibit its catalytic activity by reacting with heme radicals.

To better understand how bMDs interact with Cox15, further structural and mutational work is necessary. In addition, the development of an *in vitro* assay to directly monitor Cox15 catalytic activity would be invaluable for validating our hypothesis.

### From yeast to *T. cruzi*

Could the results obtained with the yeast model and the proposed MoA of bMD be relevant to the parasite *T. cruzi*? Several arguments point to its relevance.

CZ (and **4a**) present a potent activity against *T. cruzi* (4). CZ treatment decreases the parasite growth, oxygen consumption, and heme level. TcCox15 expressed in yeast severely increases its sensitivity to CZ (and **4a**). *T. cruzi*, like yeast, possesses a cytochrome *c* oxidase requiring heme A as an essential co-factor and a NADH-dehydrogenase (27). Thus, it is not unlikely that bMD treatment would have similar outcomes in *T. cruzi*.

However, the inhibition of TcCox15 should solely affect heme A level as observed in *T. cruzi* expressing a functionally impaired TcCox15 (17). Therefore, the effect of CZ on heme B level (Fig. 5) was unexpected.

The decrease of heme B was not observed in yeast. The source of heme B is different in the two organisms. Contrary to yeast that synthesizes its own heme B, the parasite lacks a heme B biosynthesis pathway and therefore imports its heme B from the host (27–29). CZ could impact the intracellular pool of heme B by inhibiting its uptake. Our data, however, did not support a potential effect of CZ on heme uptake as we observed a lower level of heme B upon CZ treatment in *T. cruzi* cultured both in medium with and without added hemin.

The decrease in heme B level could be the consequence of ROS overproduction induced by CZ.

As previously reported (see [4], supportive information), the synergistic combination of the anti-*T*. *cruzi* **4a** with ascorbic acid was found to increase ROS level and lipid peroxidation in *T. cruzi*, suggesting that the compounds alone could impact ROS production even though at undetectable levels in the conditions of those assays.

As a response to the increased level of ROS, the parasite might decrease the level of heme B to protect the cell components against damaging reactions, as heme B is a source of ROS (30). That response was not observed in yeast. Yeast and *T. cruzi* present key differences in their defense mechanisms against ROS; for example, the parasite lacks the catalase and the cytosolic copper-zinc superoxide dismutase Sod1 (31, 32). Thus, *T. cruzi* with a different repertoire for antioxidant defense might use alternative ways such as the degradation and/or detoxification of intracellular heme B to protect its cellular integrity against CZ-induced ROS.

Heme A derives from heme B by the sequential actions of the heme O synthase (Cox10) and the heme A synthase (Cox15). Thus, a decreased level of heme B could result in a lower level of heme A. Therefore, the impact of CZ on TcCox15 could not be proved on the basis of the data obtained with *T. cruzi* showing decreased levels of both heme B and heme A upon CZ treatment. However, the data obtained with the yeast model expressing TcCox15 strongly support an inhibitory effect of CZ on that enzyme.

An impairment of Cox15 activity by bMDs could have several consequences. In addition to the loss of cytochrome *c* oxidase, and thus of the respiratory function, bMD-induced Cox15 dysfunction could result in an increased level of heme O or the release of heme A intermediates, which with the overproduction of ROS induced by bMDs, would cause oxidative damage and might induce ferroptosis in the parasite (a process reported in *T. cruzi* [33]). Interestingly, it was recently shown that in mammalian oocytes, Cox15 defect resulted in impaired Fe^2+^ and ROS homeostasis, leading to mitochondrial dysfunction and ferroptosis (34).

**Fig 8:**
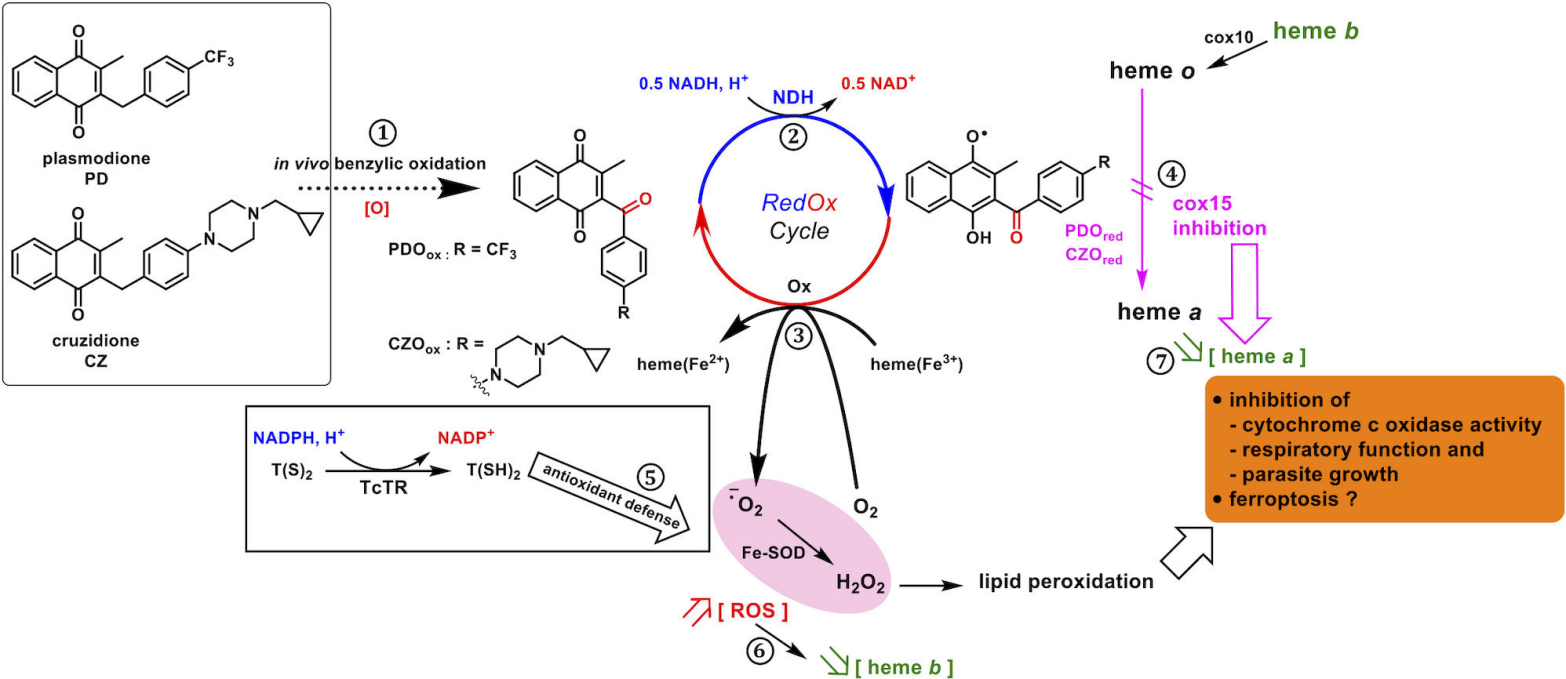
Proposed mechanism of action of bMDs in *T. cruzi*. The benzylic oxidation of parent benzylmenadiones (PD, CZ) generates the 3-benzoylmenadione metabolites (PDO, CZO) (1), which act as subversive substrates of flavoenzymes (mainly NADH-dehydrogenase (NDH) in yeast) and are reduced (2). The (1 e^-^)-reduced benzoylmenadione (PDO_red_, CZO_red_) species transfer electrons (3) to hemes/protein-bound hemes and to oxygen leading to superoxide anion radical and hydrogen peroxide production at the expense of NADH (redox-cycling process) and/or (4) interferes with Cox15 intermediate radical leading to the inhibition of the heme A synthase activity (5). The trypanothione reductase/trypanothione antioxidant system might partly protect against the bMD-induced ROS overproduction (6). The increased ROS level might result in decreased heme B level, as an attempt to protect the cells against ROS damage (7). The inhibition of Cox15 decreases heme A availability for cytochrome *c* oxidase, leading to the inhibition of respiratory function and growth. Eventually, the increased ROS level with the subsequent lipid peroxidation and the dysfunction of Cox15 activity might lead to ferroptosis.

The MoA proposed to occur in the parasite exposed to bMDs is presented in Fig. 8.

The potent and specific activity of the anti-*T*. *cruzi* bMDs against the parasite (4) is likely to be due to several factors. TcCox15 sensitivity to the compounds, as inferred from our data, might be one of the determinants. We previously reported that heme A synthase and cytochrome *c* oxidase activity were essential for *T. cruzi* infectivity and replication (17). Therefore, Cox15 seems an interesting but still under-explored druggable target for antiparasitic compounds.

Chagas disease remains a major public health issue in Latin America, with expanding risk zones due to globalization and climate change. Current treatment options are limited to two drugs, benznidazole and nifurtimox. Interestingly, both are redox-active compounds, as are other drugs that are the cornerstone of parasite chemotherapy. The development of novel drugs such as the redox-active bMDs and the finding of novel drug targets such as Cox15 are most needed to combat that neglected disease.

## SUPPLEMENTAL MATERIAL

Supplemental material aac.01161-25-s0001.pdf Fig. S1 and S2.

ASM does not own the copyrights to Supplemental Material that may be linked to, or accessed through, an article. The authors have granted ASM a non-exclusive, world-wide license to publish the Supplemental Material files. Please contact the corresponding author directly for reuse.

## References

[1] Müller T, Johann L, Jannack B, Brückner M, Lanfranchi DA, Bauer H, Sanchez C, Yardley V, Deregnaucourt C, Schrével J, Lanzer M, Schirmer RH, Davioud-Charvet E. 2011. Glutathione reductase-catalyzed cascade of redox reactions to bioactivate potent antimalarial 1,4-naphthoquinones – a new strategy to combat malarial parasites. J Am Chem Soc 133:11557–11571. doi:10.1021/ja201729z 21682307

[2] Ehrhardt K, Deregnaucourt C, Goetz AA, Tzanova T, Gallo V, Arese P, Pradines B, Adjalley SH, Bagrel D, Blandin S, Lanzer M, Davioud-Charvet E. 2016. The redox cycler plasmodione is a fast-acting antimalarial lead compound with pronounced activity against sexual and early asexual blood-stage parasites. Antimicrob Agents Chemother 60:5146–5158. doi:10.1128/AAC.02975-15 27297478 PMC4997830

[3] Bielitza M, Belorgey D, Ehrhardt K, Johann L, Lanfranchi DA, Gallo V, Schwarzer E, Mohring F, Jortzik E, Williams DL, Becker K, Arese P, Elhabiri M, Davioud-Charvet E. 2015. Antimalarial NADPH-consuming redox-cyclers as superior glucose-6-phosphate dehydrogenase deficiency copycats. Antioxid Redox Signal 22:1337–1351. doi:10.1089/ars.2014.6047 25714942 PMC4410756

[4] Trometer N, Pecourneau J, Feng L, Navarro-Huerta JA, Lazarin-Bidóia D, de Oliveira Silva Lautenschlager S, Maes L, Fortes Francisco A, Kelly JM, Meunier B, Cal M, Mäser P, Kaiser M, Davioud-Charvet E. 2024. Synthesis and anti-Chagas activity profile of a redox-active lead 3-benzylmenadione revealed by high-content imaging. ACS Infect Dis 10:1808–1838. doi:10.1021/acsinfecdis.4c00137 38606978

[5] Feng L, Lanfranchi DA, Cotos L, Cesar-Rodo E, Ehrhardt K, Goetz A-A, Zimmermann H, Fenaille F, Blandin SA, Davioud-Charvet E. 2018. Synthesis of plasmodione metabolites and ^13^C-enriched plasmodione as chemical tools for drug metabolism investigation. Org Biomol Chem 16:2647–2665. doi:10.1039/c8ob00227d 29542786

[6] Cichocki BA, Donzel M, Heimsch KC, Lesanavičius M, Feng L, Montagut EJ, Becker K, Aliverti A, Elhabiri M, Čėnas N, Davioud-Charvet E. 2021. Plasmodium falciparum ferredoxin-NADP^+^ reductase-catalyzed redox cycling of plasmodione generates both predicted key drug metabolites: implication for antimalarial drug development. ACS Infect Dis 7:1996–2012. doi:10.1021/acsinfecdis.1c00054 33855850

[7] Spolitak T, Hollenberg PF, Ballou DP. 2016. Oxidative hemoglobin reactions: applications to drug metabolism. Arch Biochem Biophys 600:33–46. doi:10.1016/j.abb.2016.04.007 27091316

[8] Mounkoro P, Michel T, Golinelli-Cohen MP, Blandin S, Davioud-Charvet E, Meunier B. 2021. A role for the succinate dehydrogenase in the mode of action of the redox-active antimalarial drug, plasmodione. Free Radic Biol Med 162:533–541. doi:10.1016/j.freeradbiomed.2020.11.010 33232753

[9] Mounkoro P, Michel T, Blandin S, Golinelli-Cohen MP, Davioud-Charvet E, Meunier B. 2019. Investigating the mode of action of the redox-active antimalarial drug plasmodione using the yeast model. Free Radic Biol Med 141:269–278. doi:10.1016/j.freeradbiomed.2019.06.026 31238126

[10] Blaza JN, Bridges HR, Aragão D, Dunn EA, Heikal A, Cook GM, Nakatani Y, Hirst J. 2017. The mechanism of catalysis by type-II NADH:quinone oxidoreductases. Sci Rep 7:40165. doi:10.1038/srep40165 28067272 PMC5220320

[11] Salmon-Chemin L, Buisine E, Yardley V, Kohler S, Debreu MA, Landry V, Sergheraert C, Croft SL, Krauth-Siegel RL, Davioud-Charvet E. 2001. 2- and 3-substituted 1,4-naphthoquinone derivatives as subversive substrates of trypanothione reductase and lipoamide dehydrogenase from Trypanosoma cruzi: synthesis and correlation between redox cycling activities and in vitro cytotoxicity. J Med Chem 44:548–565. doi:10.1021/jm001079l 11170645

[12] Ottilie S, Goldgof GM, Calvet CM, Jennings GK, LaMonte G, Schenken J, Vigil E, Kumar P, McCall L-I, Lopes ESC, Gunawan F, Yang J, Suzuki Y, Siqueira-Neto JL, McKerrow JH, Amaro RE, Podust LM, Durrant JD, Winzeler EA. 2017. Rapid Chagas disease drug target discovery using directed evolution in drug-sensitive yeast. ACS Chem Biol 12:422–434. doi:10.1021/acschembio.6b01037 27977118 PMC5649375

[13] Laleve A, Panozzo C, Kühl I, Bourand-Plantefol A, Ostojic J, Sissoko A, Tribouillard-Tanvier D, Cornu D, Burg A, Meunier B, Blondel M, Clain J, Bonnefoy N, Duval R, Dujardin G. 2020. Artemisinin and its derivatives target mitochondrial c-type cytochromes in yeast and human cells. Biochim Biophys Acta Mol Cell Res 1867:118661. doi:10.1016/j.bbamcr.2020.118661 31987792

[14] Lemaire C, Dujardin G. 2008. Preparation of respiratory chain complexes from Saccharomyces cerevisiae wild-type and mutant mitochondria: activity measurement and subunit composition analysis. Methods Mol Biol 432:65–81. doi:10.1007/978-1-59745-028-7_5 18370011

[15] Chen X, Zhang Y, Lu C, Ma W, Guan J, Gong C, Yang J, Zhang H, Zhang K, Wu S, Zhou K, Yang Y, Liu Z, Wang L, Shi B, Shi S, Xiao W, ByteDance AML AI4Science Team. 2025. Protenix - advancing structure prediction through a comprehensive AlphaFold3 reproduction. bioRxiv. doi:10.1101/2025.01.08.631967

[16] Pettersen EF, Goddard TD, Huang CC, Couch GS, Greenblatt DM, Meng EC, Ferrin TE. 2004. UCSF Chimera—A visualization system for exploratory research and analysis. J Comput Chem 25:1605–1612. doi:10.1002/jcc.20084 15264254

[17] Merli ML, Cirulli BA, Menéndez-Bravo SM, Cricco JA. 2017. Heme A synthesis and CcO activity are essential for Trypanosoma cruzi infectivity and replication. Biochem J 474:2315–2332. doi:10.1042/BCJ20170084 28588043

[18] Berry EA, Trumpower BL. 1987. Simultaneous determination of hemes a, b, and c from pyridine hemochrome spectra. Anal Biochem 161:1–15. doi:10.1016/0003-2697(87)90643-9 3578775

[19] Kolaczkowska A, Kolaczkowski M, Goffeau A, Moye-Rowley WS. 2008. Compensatory activation of the multidrug transporters Pdr5p, Snq2p, and Yor1p by Pdr1p in Saccharomyces cerevisiae. FEBS Lett 582:977–983. doi:10.1016/j.febslet.2008.02.045 18307995 PMC2288637

[20] Buchensky C, Almirón P, Mantilla BS, Silber AM, Cricco JA. 2010. The Trypanosoma cruzi proteins TcCox10 and TcCox15 catalyze the formation of heme A in the yeast Saccharomyces cerevisiae. FEMS Microbiol Lett 312:133–141. doi:10.1111/j.1574-6968.2010.02109.x 20979346

[21] Schulz V, Basu S, Freibert S-A, Webert H, Boss L, Mühlenhoff U, Pierrel F, Essen L-O, Warui DM, Booker SJ, Stehling O, Lill R. 2023. Functional spectrum and specificity of mitochondrial ferredoxins FDX1 and FDX2. Nat Chem Biol 19:206–217. doi:10.1038/s41589-022-01159-4 36280795 PMC10873809

[22] Rivett E.D, Heo L, Feig M, Hegg EL. 2021. Biosynthesis and trafficking of heme o and heme a: new structural insights and their implications for reaction mechanisms and prenylated heme transfer. Crit Rev Biochem Mol Biol 56:640–668. doi:10.1080/10409238.2021.1957668 34428995 PMC8877297

[23] Niwa S, Takeda K, Kosugi M, Tsutsumi E, Mogi T, Miki K. 2018. Crystal structure of heme A synthase from Bacillus subtilis. Proc Natl Acad Sci USA 115:11953–11957. doi:10.1073/pnas.1813346115 30397130 PMC6255202

[24] Rivett ED, Addis HG, Dietz JV, Carroll-Deaton JA, Gupta S, Foreman KL, Dang MA, Fox JL, Khalimonchuk O, Hegg EL. 2023. Evidence that the catalytic mechanism of heme a synthase involves the formation of a carbocation stabilized by a conserved glutamate. Arch Biochem Biophys 744:109665. doi:10.1016/j.abb.2023.109665 37348627 PMC10529832

[25] Butler J, Koppenol WH, Margoliash E. 1982. Kinetics and mechanism of the reduction of ferricytochrome c by the superoxide anion. J Biol Chem 257:10747–10750. doi:10.1016/S0021-9258(18)33886-9 6286671

[26] Lalève A, Vallières C, Golinelli-Cohen M-P, Bouton C, Song Z, Pawlik G, Tindall SM, Avery SV, Clain J, Meunier B. 2016. The antimalarial drug primaquine targets Fe-S cluster proteins and yeast respiratory growth. Redox Biol 7:21–29. doi:10.1016/j.redox.2015.10.008 26629948 PMC4683384

[27] El-Sayed NM, Myler PJ, Bartholomeu DC, Nilsson D, Aggarwal G, Tran A-N, Ghedin E, Worthey EA, Delcher AL, Blandin G, et al.. 2005. The genome sequence of Trypanosoma cruzi, etiologic agent of Chagas disease. Science 309:409–415. doi:10.1126/science.1112631 16020725

[28] Korený L, Lukes J, Oborník M. 2010. Evolution of the haem synthetic pathway in kinetoplastid flagellates: an essential pathway that is not essential after all? Int J Parasitol 40:149–156. doi:10.1016/j.ijpara.2009.11.007 19968994

[29] Tripodi KEJ, Menendez Bravo SM, Cricco JA. 2011. Role of heme and heme-proteins in trypanosomatid essential metabolic pathways. Enzyme Res 2011:873230. doi:10.4061/2011/873230 21603276 PMC3092630

[30] Nogueira N de A, Souza C de, Saraiva F de S, Sultano PE, Dalmau SR, Bruno RE, de Lima Sales Gonçalves R, Laranja GAT, Leal LHM, Coelho MGP, Masuda CA, Oliveira MF, Paes MC. 2011. Heme-induced ROS in Trypanosoma cruzi activates CaMKII-like that triggers epimastigote proliferation. One helpful effect of ROS. PLoS One 6:e25935. doi:10.1371/journal.pone.0025935 22022475 PMC3191175

[31] Freire ACG, Alves CL, Goes GR, Resende BC, Moretti NS, Nunes VS, Aguiar PHN, Tahara EB, Franco GR, Macedo AM, Pena SDJ, Gadelha FR, Guarneri AA, Schenkman S, Vieira LQ, Machado CR. 2017. Catalase expression impairs oxidative stress-mediated signalling in Trypanosoma cruzi. Parasitology 144:1498–1510. doi:10.1017/S0031182017001044 28653592

[32] Merli ML, Mediavilla MG, Zhu X, Cobine PA, Cricco JA. 2025. Solving the puzzle of copper trafficking in Trypanosoma cruzi: candidate genes that can balance uptake and toxicity. FEBS J 292:391–411. doi:10.1111/febs.17340 39639518

[33] Bogacz M, Krauth-Siegel RL. 2018. Tryparedoxin peroxidase-deficiency commits trypanosomes to ferroptosis-type cell death. eLife 7:1–29. doi:10.7554/eLife.37503 PMC6117152 30047863

[34] Zhang Z, Yu R, Shi Q, Wu ZJ, Li Q, Mu J, Chen B, Shi J, Ni R, Wu L, Li Q, Fu J, Li R, Sun X, Wang J, He L, Kuang Y, Sang Q, Wang L. 2024. COX15 deficiency causes oocyte ferroptosis. Proc Natl Acad Sci USA 121:e2406174121. doi:10.1073/pnas.2406174121 39471219 PMC11551447

