# Supplementary material for: The heme A synthase Cox15, as a target of redox-active 3-benzylmenadiones with antiparasitic activity
Source: Antimicrob Agents Chemother. 2025 Dec 10;70(1):e01161-25. doi: 10.1128/aac.01161-25 (PMC12777560; doi:10.1128/aac.01161-25)
Supplement: Supplemental material — Fig. S1 and S2. [file aac.01161-25-s0001.pdf]

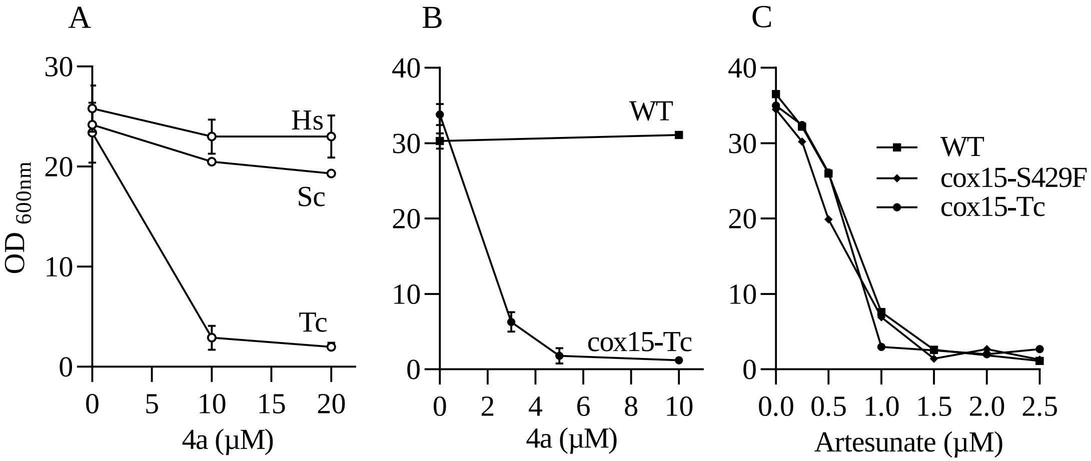

Fig.S1A) Growth sensitivity to **4a** of mutants expressing exogenous *COX15*. Mutant Δcox15 was transformed with a plasmid harbouring yeast *COX15* (control, Sc) or COX15 from the two other species under the control of yeast *COX15* promoter and terminator, *T. cruzi* (Tc) and *H. sapiens* (Hs); B) Growth sensitivity to **4a** of mutant cox15-Tc expressing of *T. cruzi* COX15 ORF that replaces yeast COX15 ORF at the genomic locus. C) Growth sensitivity to artesunate of WT, cox15-S429F and cox15-Tc. The cells were grown in YPEth medium with various concentrations of drugs. OD_{600nm} were measured after three days. A and B) The values are means ±SD of two replicate measurements.

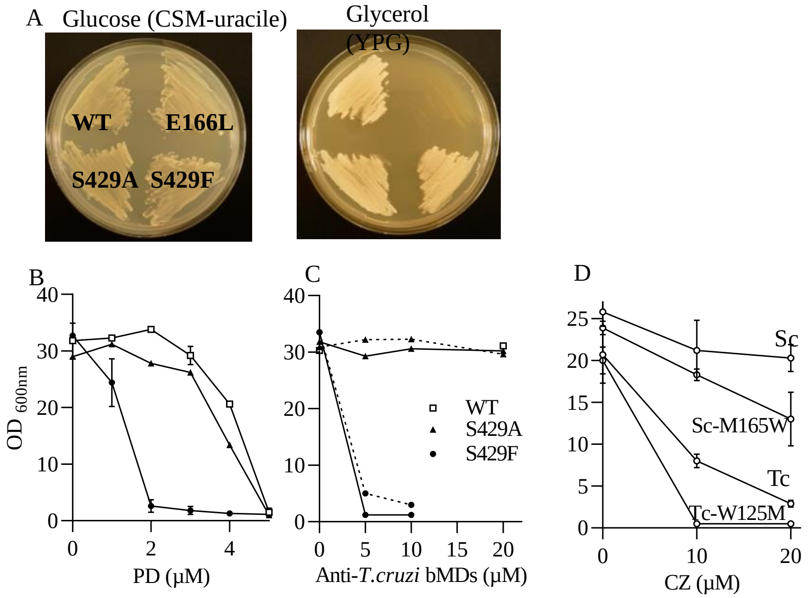

Fig.S2 Effect of mutations in Cox15. A) Respiratory growth competence. *Δcox15* cells transformed with a plasmid bearing WT or mutated *COX15* were inoculated on glucose plate (CSM-uracil medium, selective for the plasmid) and on glycerol plate (YPG). The plates were observed after four day incubation; B and C) Sensitivity to bMDs. WT and mutants (harbouring cox15-S429A or cox15-S429F mutation at the genomic locus) were grown in YPEth medium with various concentrations of: B) PD and C) 4a (straight lines) or CZ (dotted lines). OD_{600nm} were measured after three days; D) Sensitivity to CZ. Yeast *Δcox15* were transformed with a plasmid harbouring yeast *COX15* (Sc), yeast *COX15* with the mutation M165W (Sc-M165W), *COX15* from *T.cruzi* (Tc) or *T.cruzi COX15* with the mutation W125M (Tc-W125M). The cells were grown in YPEth medium with various concentrations of CZ. OD_{600}nm were measured after three days. (B, D) The values are means ±SD from two replicate measurements.
